# What Makes Babies Musical? Conceptions of Musicality in Infants and Toddlers

**DOI:** 10.3389/fpsyg.2021.736833

**Published:** 2021-12-15

**Authors:** Verena Buren, Daniel Müllensiefen, Tina C. Roeske, Franziska Degé

**Affiliations:** ^1^Department of Music, Max Planck Institute for Empirical Aesthetics, Frankfurt/M, Germany; ^2^Department of Psychology, Goldsmiths, University of London, London, United Kingdom

**Keywords:** musicality, musical ability, conceptions of musical ability, development of musical ability, survey, infants, toddlers, musical development

## Abstract

Despite major advances in research on musical ability in infants, relatively little attention has been paid to individual differences in general musicality in infants. A fundamental problem has been the lack of a clear definition of what constitutes “general musicality” or “musical ability” in infants and toddlers, resulting in a wide range of test procedures that rely on different models of musicality. However, musicality can be seen as a social construct that can take on different meanings across cultures, sub-groups, and individuals, and may be subject to change over time. Therefore, one way to get a clearer picture of infant musicality is to assess conceptions of musicality in the general population. Using this approach, we surveyed 174 German adults, asking about their view and conceptions regarding behaviors that characterize a musical child under 3 years. Based on previous studies on adult and child musicality, we designed a survey containing 41 statements describing musical behaviors in children. Participants were asked to rate how indicative these behaviors were of musicality in infants and toddlers. PCA analysis revealed 4 components of musical abilities and behaviors in under-3-year-olds: Musical Communication, Enthusiasm and Motivation, Adaptive Expressiveness, and Musical Abilities as traditionally defined. Professional background and musical expertise of the respondents did not significantly influence participants’ conceptions. Our results suggest that, in order to capture musicality in young children, a wider range of skills and observable behaviors should be taken into account than those assessed by traditional musical ability tests for young children.

## Introduction

Across the last century, a wide range of tests have been developed to measure musical ability in childhood (e.g., Gordon, 1965, 1979, 1982, 1989a,b; Bentley, 1966; Seashore, 1967; Wing, 1981). Strikingly, these instruments are based on a very diverse range of different theoretical models and conceptions of musical ability (Shuter-Dyson, 1999). This diversity is due to the fact that musicality is not a natural category, but a social construct that can take on different meanings in different cultures, subgroups, and even individuals (Blacking, 1971; Hallam and Prince, 2003). We here use the term musicality to describe a broader understanding of musical ability that includes not only musical perception and production, but also emotional responses to music, emotional expressiveness through music, and interest and motivation in musical activities. In contrast to the term musicality, the term musical ability is often associated with musical perception or production skills assessed by traditional behavioral tests. The social construct of musicality cannot be assessed directly because it can manifest itself through a wide range of observable behaviors with relation to music. Therefore, it is necessary to identify indicators that reliably reflect the construct. The identification of behavioral indicators of musicality can lead to a broader understanding of how (conceptions of) musicality change(s) across different target ages and serve as a starting point for the development of age-appropriate test procedures, which can then be used to determine early musical skills and to map individual developmental trajectories.

In order to investigate musical development from the very beginning, it is essential to capture the first signs of musicality. Early observations by parents and educators can provide important information about which behaviors are perceived as the first indicators of musicality. Therefore, the objective of the present study was to investigate conceptions of infant/toddler musicality as perceived by adults in Germany with different professional backgrounds and musical expertise. The aim was to determine which abilities and behaviors in infants and toddlers are interpreted as indicators of musicality. This knowledge will enable us to deepen our understanding of what constitutes musicality in the first years and to develop a clearer picture of early musicality and its facets. The results of this study may help to explain how musicality can be described in infants and toddlers and thus provide a basis for developing appropriate measurement procedures in the future.

### The Development of Musical Abilities

Music is a cultural universal (Merriam, 1964; Blacking, 1995) and all humans are assumed to possess a potential for musical competence (Trehub et al., 2015a). Current research suggests that the development of musicality begins in the womb and continues into adulthood (Gooding and Standley, 2011) through processes of enculturation and musical training (Hannon and Trainor, 2007).

#### Musical Development in the First Years of Life

Already in the first months of life, children can develop amazing perceptual abilities (Trehub, 2003). For example, sensitivity to melodic contours and relative pitches are developed in early infancy (Trehub, 2015). Moreover, certain perceptual abilities that are central to music cognition are initially universal, i.e., infants have the perceptual prerequisites to recognize the musical subtleties of any musical culture (Trehub, 2015). However, over time, culture-specific attunement takes place, and children develop more and more culture-specific skills (Trehub, 2015). An example is rhythm perception, where 6-month-old infants show a culture-general pattern of responding to musical rhythms, whereas 12-month-old infants already show an adult-like, culture-specific pattern of responding (Hannon and Trehub, 2005).

Rhythm production skills also develop rapidly in early childhood. Although two-and-a-half-year-olds can already adapt the tempo of their drumming to that of a drumming partner (Kirschner and Tomasello, 2009), synchronous drumming with a rhythmic pattern is not achieved until around the age of 4 (Provasi and Bobin-Bègue, 2003). Other skills (such as sensitivity to harmony) do not develop until later childhood (e.g., Schellenberg et al., 2005).

Infants are also generally attracted to music and music-like stimuli (like infant-directed speech) from a very early age and give carefully matched expressive responses. By doing this, they create co-operative patterns of communication that can be interpreted as a form of communicative musicality (Malloch, 2000; Flohr and Trevarthen, 2008), in which early singing-like and speaking-like vocalizations are still indistinguishable (Stadler Elmer, 2012). After their first birthday, children are increasingly able to express themselves musically: they dance to music in a rudimentary way (Trehub, 2015), and a few months later, their early vocalizations differentiate into speaking and singing, with early singing showing characteristic features such as glissandi, unstable pitches, singing with indefinable sounds, neologisms, short phrases in a narrow vocal range, and small and imprecisely tuned intervals (Stadler Elmer, 2012). Thus, while rudimentary singing is already present in under 3-year-olds, children become more sophisticated between the ages of 3 and 4 and begin to combine songs or song fragments. They hereby increasingly succeed in approximating the phrase contours of a song (Gembris, 2017), until the development of singing ability is largely complete in 8-year-olds (Davidson, 1994).

#### The Musical Environment of Babies

Especially in early childhood, parents and their musical practices in the home environment have a major influence on children’s musical lives (Ilari, 2018). One of the first and most common musical interactions with babies is singing (Ilari, 2005; Young, 2008). When singing to their infants, parents sing in a distinctive manner [e.g., by using high pitch, slow tempo, and more expressive rendering of lyrics (Trehub et al., 1997a,b)]. This so-called infant-directed singing is often used to modulate infant arousal (Shenfield et al., 2003; Trehub et al., 2015b) and it also serves social bonding functions (Trehub, 2015). Especially in musical interactions with babies, singing is an important way of musical interaction with the caregiver (Ilari, 2005; Costa-Giomi and Ilari, 2014). Additionally, the special importance of the voice has also been demonstrated in a series of studies which have shown that sung melodies are better remembered than instrumental melodies (Weiss et al., 2012, 2015, 2021). Besides singing, many parents rely on recorded music or modern toys with musical features to create a musical environment for their children (de Vries, 2009; Mehr, 2014). Although the use of recorded music offers many advantages (e.g., the ability to engage with music without a parent, the ability to offer a greater variability of styles), recorded music lacks the multimodality of live interaction with a caregiver. In addition, there seems to be a widespread belief that educational settings provide children with a more comprehensive musical experience (de Vries, 2009) which may lead parents to think that their own musical interactions are of lesser importance. This suggests that parents’ goals and beliefs about their children’s musical development are very influential, as they affect the styles and practices they use with their children (McPherson, 2009). Therefore, it is important to define musical development not only from current theories of human development, but also to consider musicality as a social construct that is influenced by contemporary discourses in music education research, by different approaches in educational practice, and by common believes about child musicality as held by parents, carers, and educators.

### Assessing Musicality in Childhood

There are already many findings on “age-related stages of mastery of basic elements of the Western-European music system” (Stadler Elmer, 2011, p. 13). Thus, many studies examine skills that are present on average at a given age in order to draw conclusions about common developmental trajectories (Stadler Elmer, 2011). However, this can lead to glossing over or omitting important dimensions/indices of musical development (Forrester and Borthwick-Hunter, 2015). Although this approach may show that a skill or competence has emerged, it can easily fail to capture the causes, context and important associations of its emergence (Forrester and Borthwick-Hunter, 2015). A more comprehensive approach requires the consideration of more complex behaviors in culturally embedded settings (as has been shown for early singing development; see Stadler Elmer, 2011, 2012). The extension and integration of the existing findings and the implementation of a test battery based on these findings to monitor musical developmental processes could be a long-term goal of research on musical development.

While developmental science has made great progress in assessing how and when different basic musical abilities develop (see Trehub and Hannon, 2006; Trainor and Unrau, 2011; Trehub, 2015, for comprehensive reviews on this topic), relatively little progress has been made toward understanding how these abilities relate to each other and to the conception of general musicality. To study the development of general musicality adequately, we need scientific measurement tools that have a solid basis in developmental theory as well as being compatible with the conceptions of musicality of important stakeholders (i.e., general and music educators, carers, parents).

One approach to the study of musical ability is the use of standardized tests. However, these cannot be used with children under 3 years of age. Additionally, the traditional tests used in later childhood [such as the Seashore Measures of Musical Talent (Seashore, 1919), the Measures of Musical Abilities developed by Bentley (1966), or the “Gordon tests” (Gordon, 1965, 1979, 1982, 1989a,b)] place an emphasis on auditory and receptive skills and largely neglect music production behavior (composing, improvising, playing an instrument, or singing), motivation, and emotional and communicative elements (such as aesthetic responses, musical communication, and expressiveness). Thus, these measures are unlikely to be sufficient to provide a comprehensive picture of musicality and its development (Murphy, 1999). In addition, before assessing a construct, it is useful to clearly define which components are to be assessed in the first place (which requires a clear definition of musicality and its different facets). Some researchers have even questioned whether a single test of musical ability can capture the manifold manifestations of musicality at all (Murphy, 1999), which is especially true for infants and young children. Therefore, measurement instruments need to be highly adapted or supplemented with age-appropriate observational instruments based on a clear definition of musicality.

Parent reports can therefore be a valuable source for the study of musical behaviors in young children. An example is the Children’s Musical Behavior Questionnaire (Valerio et al., 2012), which surveys music-related behaviors (as documented by parents about children and themselves). It includes 97 items aimed at finding out how often children exhibit a range of musical behaviors (e.g., singing, dancing, or listening to music). Seven factors relate to child-initiated musical behaviors (Attention and Emotion, Vocalizations, Moving, Daily Routines, Requests, Taking Turns, Creativity) and one relates to parent-initiated activities.

An even more comprehensive survey is the Music@home questionnaire (Politimou et al., 2018) which was designed to systematically map musical engagement in the home environment of young children. The infant version (3–23 months) of the Music@Home scale examines four factors: Parental beliefs (i.e., what parents think about music and development), Child engagement with music, Parent initiation of singing, and Parent initiation of music-making. In the Preschool version (2–5.5 years) the four factors were Parental beliefs, Child engagement with music, Parent initiation of singing, and Breadth of musical exposure.

Questionnaires such as the Children’s Musical Behavior Questionnaire and the Music@home questionnaire are valuable for identifying musical opportunities and behaviors in infants and young children. They enable the empirical documentation of musical behaviors that children exhibit in their home environment and how parents use their musical nurturing opportunities. In addition, they take advantage of primary caregivers’ rich knowledge about their children’s development, which cannot always be adequately accounted for in laboratory studies. However, they do not extensively elucidate what *conceptions* of musicality underlie the behaviors exhibited. Moreover, interindividual differences and comparisons across different trajectories of musical development are much less a focus of these questionnaire and survey instruments.

### Conceptions of Musicality

Approaching musicality as a social construct, Hallam and colleagues examined how people with different musical experiences conceptualize musicality (Hallam and Shaw, 2002; Hallam and Prince, 2003; Hallam, 2010; Hallam and Papageorgi, 2016). In a first qualitative study, Hallam and Prince (2003) asked 415 participants (adults and adolescents) from the United Kingdom to complete in writing the statement “musical ability is…”. In an iterative process of categorization, the collected statements were analyzed and six overarching themes were identified (aural abilities, receptive responses, generative activities, integration of a range of abilities, personal characteristics, whether musical abilities are innate or learned). Overall, the statements contained more references to active music making than to receptive skills and became more complex as respondents’ musical experience increased.

The 77 statements derived from the qualitative study then served as a starting point for the quantitative study of musical conceptions (Hallam, 2010). Six hundred and sixty participants (musicians, educators, amateur musicians, children with and without musical engagement) rated these statements according to their level of agreement. Through principal component analysis, the authors identified six components of musicality: (1) playing an instrument or singing, (2) musical communication (communicating emotions through music), (3) valuing, appreciating, and responding to music, (4) composition, improvisation, and related skills, (5) commitment, motivation, personal discipline and organization, (6) rhythmic ability, pitch, and understanding. The conceptions differed between participants depending on their professional and musical experiences: Musical communication was perceived as the most important ability by musicians, whereas educators placed more emphasis on creativity. In contrast, amateur musicians and non-musicians held the belief that musicality is constituted by high aural skills and motivation.

In order to investigate whether these conceptions of musicality in general also apply to conceptions of musicality in childhood, the statements of the original study by Hallam and Prince (2003) were adapted so that they corresponded in principle to the abilities of the relevant age group (in this case, 3- to 6-year-olds; Buren et al., 2021). Nine hundred and twenty-two adults in Germany (music educators and musicians, educators but without specialization in music, parents and carers) rated the 49 resulting statements according to how frequently they thought a musical child between the ages of 3 and 6 exhibited these behaviors. Similar to Hallam’s (2010) results, a differentiated and multifaceted picture of musicality emerged. Through PCA analysis, four components of children’s musical abilities and behaviors were identified: (1) Musical Communication, (2) Enthusiasm and Motivation (the affinity and the enjoyment of music and the motivation to make music), (3) Analytical Understanding (the awareness of different aspects of music and the use of this knowledge to evaluate music), and (4) Musical Abilities (audiation abilities and the integration of these abilities for making music). On average, participants rated enthusiasm and motivation as the most important indicators of musicality in childhood, followed by musical abilities like audiation skills. Musical and educational training of the survey participants only affected the ratings on the component Analytical Understanding. The analytical understanding of 3- to 6-year-olds was considered more important by parents and carers than by educators, while music educators and musicians attributed the least importance to this component.

Since children under the age of 3 years have specific cognitive, motor, and emotional prerequisites (Bukatko and Daehler, 2004; Siegler et al., 2014; Berk, 2018), it remains unclear whether the conceptions of musicality in childhood (between 3 and 6 years) extend to children under the age of 3 years. Hence, it seems highly likely that adults would assess musicality in children under 3 years of age according to differing criteria.

For example, in terms of the component Musical Communication, it is unclear whether it is seen as equally important in children under 3 years of age, because musical vocalizations before 2 years of age are hard to distinguish from preverbal vocalizations (Stadler Elmer, 2012). Since the components Analytical Understanding and Musical Abilities are also heavily influenced by basic cognitive and linguistic skills, it remains to be seen whether they are also considered important for conceptions of musicality in children under 3 years. Furthermore, the component of musical abilities may also play a different role in younger children, since musical production skills in particular (e.g., tapping, drumming) are strongly dependent on age-related motor development processes.

The aim of this study was to draw a more comprehensive picture of infant musicality which will allow us to develop meaningful, reliable, and objective test procedures for musicality that build on a clear operational definition of the construct being measured. In addition to approaching musicality from a theoretical perspective, the empirical approach pursued in this study can confirm and complement assumptions derived from theories on musical development (e.g., Swanwick and Tillman, 1986; Serafine, 1988; Hargreaves and Galton, 1992; Gordon, 2007; Ockelford, 2013). The everyday experience of parents and educators can provide added value that can serve to refine theories. Theoretical approaches, such as general factor or multifactorial approaches, serve to systematize in a meaningful way, but should also stand up to empirical scrutiny.

In the present study, we investigated how German adults from different professional backgrounds and with a range of musical expertise perceive the construct musicality in early childhood. More specifically, we were interested in their assessment of behavioral indicators and personal factors that might be considered expressions of musicality. To this end, we created a questionnaire that contained statements about musical abilities and behaviors and asked participants with varying professional and musical backgrounds to rate these statements, according to how well they thought the statement described musicality in under-3-year-olds. The results of this study can serve as a basis for the development of a musicality test that does not reduce musicality to individual aspects, but reflects a more comprehensive understanding of musical behavior.

## Materials and Methods

### Participants

The sample comprised 221 participants between 20 and 69 years (*M* = 38.97, *SD* = 8.89) who indicated that they regularly spend time with children under the age of 3 years. Participants were excluded if they had filled in less than 50% of the questionnaire items (*n* = 29), if they had given constant ratings to all items (*n* = 9), or if they took less than 4 minutes to complete the entire survey (*n* = 9), resulting in a sample of 174 valid cases. Out of those, 146 were female and 66 had a university degree (of which *n* = 21 had a degree in music), 50 had the equivalent of A-levels, 50 had the equivalent of a general secondary school leaving certificate and 8 had a lower secondary school leaving certificate or no certificate at all. The final sample included 76 early childhood educators and 96 parents and carers without professional training in pedagogy or music education. Two participants did not indicate their profession.

### Materials

#### The Musical Child Questionnaire for Infants and Toddlers (MCQ_U3)

The Musical Child Questionnaire for children under 3 years (MCQ_U3) is an adapted version of the Musical Child Questionnaire (MCQ), which was developed to assess conceptions of musicality in children between 3 and 6 years of age (Buren et al., 2021). The basis for the development of both questionnaires was a survey by Hallam and Prince (2003) where participants had to complete the sentence: “musical ability is…” Statements were translated into German and adapted for the use with children from 0 to 3 years by adjusting to the ability spectrum of this age group. For example, the phrase “being able to move in time with a rhythm” (Hallam and Prince, 2003) was transformed to “A musical child under 3 years is able to move according to the music.” All decisions regarding the reformulation or elimination of items were made by consensus of all authors. The final version of the Musical Infant/Toddler Questionnaire consisted of 41 statements describing musical abilities and behaviors in infants and toddlers. Participants rated on a 5-point-Likert-scale (ranging from 1 = rarely/never to 5 = always) how often a child under the age of 3 years that they considered as “musical” would typically show these behaviors.

#### The Goldsmiths Musical Sophistication Index

To assess the musical background of all survey participants, they were asked to complete the seven-item musical training subscale of the Goldsmiths Musical Sophistication Index (Gold-MSI; Müllensiefen et al., 2014) in its German version (Schaal et al., 2014).

#### Demographic Information

All participants provided information about their age, gender, educational background, and profession.

### Procedure

Data were collected between April 2019 and June 2020 in Germany. Most of the respondents were recruited online using e-mail distribution lists from music schools, day care centers and the panel of a market research agency. The study was approved by the Ethics Council of the Max Planck Society, and informed consent was obtained from all participants.

### Analytic Strategy

Data analyses were performed using the R software environment (R Core Team, 2019), including the R packages psych (Revelle, 2020) and lavaan (Rosseel, 2012).

The main goal of the data analyses was to identify distinguishable facets of musical abilities and behaviors in infants/toddlers, and to compare these to the facets of musical behaviors previously identified in children between 3 and 6 years (Buren et al., 2021). As a first step, the grouping structure of the data was explored by computing a series of principal component analyses (PCA) and comparing solutions to the model previously established for children from 3 to 6 years by confirmatory factor analysis.

In a second step, a shorter scale of the MCQ_U3 with better psychometric properties was constructed using PCA. Differences in scale scores were then investigated with respect to participants’ levels of musical or educational training through correlations and multivariate analysis of variance.

## Results

### Descriptive Analysis

Table 1 lists the top 10 items that were rated by the participants as the most frequent indicators of musicality in children under 3 years of age. Interestingly, the seven items with the highest mean values are similar in content. These statements describe a high interest in music, enjoyment of music and the motivation to become musically active. The three remaining items in the top 10 list describe that a musical child has individual preferences in music taste and a “musical ear.”

**TABLE 1:** Highest rated items of the Musical Infant/Toddler Questionnaire (MCQ_U3).

Item no.	A child under 3 years who is musically skilled…	*M*	*SD*
43_U3	… enjoys the occupation with music.	4.20	0.78
38_U3	… is interested in music.	4.16	0.85
41_U3	… has great enthusiasm for music.	4.09	0.81
14_U3	… enjoys music in his/her life, either by making music or by listening to it.	4.05	0.87
45_U3	… has a great affinity for music.	3.94	0.84
15_U3	… enjoys music and appreciates sounds.	3.88	0.89
29_U3	… has the desire to make music together with others so that they become a group–either by singing along or moving to the music of others.	3.76	0.99
42_U3	… has a will of his/her own when listening and making music.	3.70	0.98
21_U3	… knows which music he/she likes and which he/she does not like.	3.70	1.08
1_U3	… has a musical ear, which means he/she can recognize simple melodies and tone progressions.	3.70	0.90

### Grouping Items of the MCQ_U3

We computed three PCA models to group the items of the MCQ_U3 into different components. The first grouping was computed following Hallam’s (2010) analysis strategy, retaining all PCA components with Eigenvalues above 2 and applying varimax rotation. The resulting model comprised 2 components and explained 44% of the variance (see Table 2 for the root mean square of the residuals). As an alternative, parallel analysis (Horn, 1965) was used to determine the number of PCA components which generated a 2-component solution to which oblimin rotation was applied. The resulting model also explained 44% of variance. Last, we specified a 4-component model with oblimin rotation to investigate similarities to the model previously established for children from 3–6 years (Buren et al., 2021). This solution explained 52% of the item variance.

**TABLE 2:** Fit indices of computed models.

	BIC	TLI	CFI	RMSEA	SRMR
2-component PCA (following Hallam, varimax rotated)	17520.93	0.778	0.790	0.074	0.070
2-component PCA (parallel analysis, oblimin rotated)	17518.47	0.778	0.790	0.074	0.070
4-component PCA (according to grouping in 3–6-year-olds, oblimin rotated)	17568.80	0.767	0.781	0.076	0.074
**4-component PCA (parallel analysis, under-3-year-olds, oblimin rotated)**	**17481.65**	**0.793**	**0.805**	**0.072**	**0.070**

*BIC, Bayesian Information Criterion; TLI, robust Tucker-Lewis Index; CFI, robust comparative fit index; RMSEA, robust root mean square error of approximation; SRMR, standardized root square residual.*

*The selected model is displayed in bold.*

All three models were compared using confirmatory factor analysis along with the previously established model for 3–6-year-olds (Buren et al., 2021). Fit indices are shown in Table 2.

Both 2-component-solutions were highly similar in content as well as in their fit indices. The 4-component PCA model proved to have the best model-data-fit, according to the Bayesian information criterion (BIC). Because both the relative (TLI, CFI) and absolute fit indices (RMSEA, SRMR) for this model were also in an acceptable to good range, we decided to select the 4-component-model for content interpretation (see Table 3 for items and their loadings).

**TABLE 3:** Items of the Musical Infant/Toddler Questionnaire (MCQ_U3), grouped by components.

	MCQ_U3 item			Component loading			
Item no.	A child under 3 years who is musically skilled…	*M*	*SD*	1	2	3	4
**Component 1: Musical Communication**							
2_U3	… can sing simple melody pieces.	3.47	1.09	0.57	0.41	–0.17	0.05
30_U3	… can invent melodies or rhythms, either with the voice, or by creating other sounds.	3.31	1.08	0.53	0.11	0.26	0.03
3_U3	… is able to internalize simple sound sequences.	3.44	0.96	0.47	0.11	0.08	0.36
8_U3	… shows that he/she can capture patterns when dancing or making music.	3.32	0.10	0.47	0.11	0.05	0.36
27_U3	… can communicate with others through music by producing musical sounds, listening, improvising, dancing, and understanding music.	3.24	1.05	0.40	0.13	0.36	0.07
**Component 2: Enthusiasm and Motivation**							
41_U3	… has great enthusiasm for music.	4.09	0.81	0.03	0.85	0.04	–0.14
38_U3	… is interested in music.	4.16	0.85	–0.08	0.75	0.07	0.08
43_U3	… enjoys the occupation with music.	4.20	0.78	0.04	0.75	0.04	–0.04
14_U3	… enjoys music in his/her life, either by making music or by listening to it.	4.05	0.87	0.07	0.72	–0.24	0.22
39_U3	… often has the desire to make music.	3.67	0.93	0.01	0.67	0.10	0.02
45_U3	… has a great affinity for music.	3.94	0.84	–0.11	0.60	–0.06	0.35
15_U3	… enjoys music and appreciates sounds.	3.88	0.89	0.05	0.56	0.18	0.03
31_U3	… likes to spontaneously produce music or musical sounds.	3.68	0.93	0.20	0.56	0.21	0.04
29_U3	… has the desire to make music together with others so that they become a group – either by singing along or moving to the music of others.	3.76	0.99	–0.07	0.51	–0.21	0.35
42_U3	… has a will of his/her own when listening and making music.	3.70	0.98	0.23	0.51	0.15	–0.03
10_U3	… actively incorporates music into his/her world.	3.49	0.98	0.24	0.50	0.27	–0.02
25_U3	… plays music with feeling.	3.57	1.08	0.30	0.47	0.16	0.01
32_U3	… is creative when making music.	3.57	1.03	0.38	0.42	0.18	–0.07
9_U3	… recognizes simple structural characteristics of music (e.g., loud–soft, slow–fast).	3.64	0.93	0.40	0.41	0.03	0.08
21_U3	… knows which music he/she likes and which he/she does not like.	3.70	1.08	0.28	0.31	0.04	0.10
**Component 3: Adaptive Expressiveness**							
44_U3	… would rather express themselves through music than through other means.	2.58	1.06	0.06	0.00	0.74	–0.02
17_U3	… appreciates different kinds of music.	3.08	1.01	–0.18	0.18	0.65	0.13
26_U3	… perceives the basic mood or feelings conveyed by the music that he/she is listening to.	3.10	0.99	–0.02	0.08	0.64	0.21
11_U3	… is able to react to the mood of a melody.	3.22	1.03	0.06	0.03	0.64	0.23
22_U3	… is able to hear differences between different types of music.	3.04	1.07	0.23	–0.06	0.56	0.14
46_U3	A child is musical when music means something to him/her.	3.23	1.12	–0.37	0.32	0.51	0.08
16_U3	… has an open mind with which he/she approaches and experiences all music.	3.40	1.00	–0.06	0.48	0.51	–0.23
18_U3	… recognizes different types of music (e.g., children’s songs, pop, classical music).	2.77	1.22	0.28	–0.20	0.49	0.09
13_U3	… is able to react creatively, emotionally and intellectually when listening to a piece of music.	3.24	1.04	0.35	0.08	0.47	0.06
19_U3	… reacts consciously to different aspects of music.	3.26	1.03	0.39	–0.12	0.46	0.15
47_U3	… can immerse him-/herself in sounds.	3.11	1.08	0.20	0.31	0.38	0.13
**Component 4: Musical Abilities**							
6_U3	… has a sense of timing and rhythm.	3.41	1.04	0.14	–0.04	–0.01	0.75
24_U3	… has good overall physical coordination.	3.19	0.91	–0.11	0.00	0.08	0.69
36_U3	… has a wide range of different skills.	3.35	0.96	–0.08	–0.10	0.28	0.58
12_U3	… is able to move according to the music.	3.51	0.93	–0.15	0.33	0.11	0.47
7_U3	… has a feeling for the beat.	3.26	1.01	0.22	0.00	0.22	0.46
1_U3	… has a musical ear, which means he/she can recognize simple melodies and tone progressions.	3.70	0.90	0.37	0.09	0.09	0.45
35_U3	… is able to combine hearing and sound production.	3.68	0.88	0.17	0.02	0.18	0.42
4_U3	… has good hearing ability.	3.63	0.90	–0.04	0.23	0.21	0.42
20_U3	… likes listening to music and finds matching gestures or movements.	3.63	0.88	0.06	0.29	0.07	0.40
5_U3	… has a good memory for patterns.	3.28	0.98	0.17	0.29	0.18	0.34

The first component included items that reflected types of musical communication (e.g., “A child under 3 years who is musically skilled can communicate with others through music by producing musical sounds, listening, improvising, dancing, and understanding music”), or musical understanding (e.g., “A child under 3 years who is musically skilled shows that he/she can capture patterns when dancing or making music”) or the ability to express oneself through music (e.g., “A child under 3 years who is musically skilled can invent melodies or rhythms, either with the voice, or by creating other sounds”). This component included factor loadings ranging from 0.40 to 0.57 and was termed Musical Communication.

The second component contained 15 items with loadings ranging from 0.31 to 0.85. We named this component Enthusiasm and Motivation, because many high loading items described general interest in music (e.g., “A child under 3 years who is musically skilled has great enthusiasm for music”) and the inherent motivation to become musically active (e.g., “A child under 3 years who is musically skilled often has the desire to make music”). Also associated with this component were items on creativity, playing with feeling, and having one’s own taste in music.

Component 3 was interpreted as Adaptive Expressiveness. This component described the ability of a musical child to respond flexibly to different types of music, to appreciate different kinds of music and to react correspondingly in creative, emotional, and intellectual ways. It comprised factor loadings from 0.38 to 0.74.

Component 4 was composed of 10 items (component loadings ranged from 0.34 to 0.75) focusing on musical abilities in a more specific sense (e.g., having a musical ear and a sense of timing, rhythm, and beat). It also contained items describing a set of more global abilities and behaviors (e.g., “A child under 3 years who is musically skilled has a wide range of different skills”; “…is able to combine hearing and sound production”). Therefore, we named the component Musical Abilities. Table 4 shows the mean ratings and the amount of explained variance by component.

**TABLE 4:** Mean ratings grouped by components.

	*M*	*SD*	*n*	Number of items included	Explained variance (%)
Musical Communication	3.36	0.77	174	5	9
Enthusiasm and Motivation	3.81	0.64	174	15	18
Adaptive Expressiveness	3.12	0.72	174	11	14
Musical Abilities	3.47	0.62	174	10	11

*SD, Standard Deviation; n, number of participants.*

### Constructing the MCQ_U3 Short Scale

Grouping the items of the MCQ_U3 provided an informative classification suggesting four different facets of young children’s musicality. In a next step, we constructed a short scale of the MCQ_U3 to obtain a more robust and practical measurement instrument for use in future studies.

The short scale was constructed using parallel analysis with PCA and subsequent oblimin rotation. Then, only items with component loadings of >0.4 were retained in order to reduce the number of items and maximize their discriminatory power. This resulted in a 2-component solution, which included 26 items and explained 51% of the variance (see Table 5 for the retained items and their respective loadings). The first component comprised a diverse set of items on perceptual and productive musical abilities and was therefore named Basic Musical Abilities (it contained items from the 3 components Musical Communication, Adaptive Expressiveness, and Musical Abilities of the 4-component solution). The second component included items on motivation, interest, and enthusiasm. Therefore, we kept the name Enthusiasm and Motivation.

**TABLE 5:** Short scale of the Musical Infant/Toddler Questionnaire grouped by components.

	MCQ_U3 item	Component loading	
		
Item no.	A child under 3 years who is musically skilled…	1	2
**Component 1: Basic Musical Abilities**			
19_U3	… reacts consciously to different aspects of music.	0.78	–0.19
22_U3	… is able to hear differences between different types of music.	0.75	–0.11
11_U3	… is able to react to the mood of a melody.	0.74	–0.02
3_U3	… is able to internalize simple sound sequences.	0.72	0.03
8_U3	… shows that he/she can capture patterns when dancing or making music.	0.68	0.04
7_U3	… has a feeling for the beat.	0.67	–0.05
13_U3	… is able to react creatively, emotionally and intellectually when listening to a piece of music.	0.67	0.04
26_U3	… perceives the basic mood or feelings conveyed by the music that he/she is listening to.	0.67	0.04
1_U3	… has a musical ear, which means he/she can recognize simple melodies and tone progressions.	0.66	0.06
27_U3	… can communicate with others through music by producing musical sounds, listening, improvising, dancing, and understanding music.	0.65	0.09
30_U3	… can invent melodies or rhythms, either with the voice, or by creating other sounds.	0.65	0.04
47_U3	… can immerse him-/herself in sounds.	0.58	0.25
5_U3	… has a good memory for patterns.	0.56	0.22
9_U3	… recognizes simple structural characteristics of music (e.g., loud–soft, slow–fast).	0.42	0.33
**Component 2: Enthusiasm and Motivation**			
41_U3	… has great enthusiasm for music.	–0.11	0.90
14_U3	… enjoys music in his/her life, either by making music or by listening to it.	–0.10	0.82
38_U3	… is interested in music.	0.03	0.77
43_U3	… enjoys the occupation with music.	0.00	0.75
39_U3	… often has the desire to make music.	0.04	0.69
45_U3	… has a great affinity for music.	0.05	0.68
15_U3	… enjoys music and appreciates sounds.	0.19	0.56
31_U3	… likes to spontaneously produce music or musical sounds.	0.34	0.55
10_U3	… actively incorporates music into his/her world.	0.37	0.48
42_U3	… has a will of his/her own when listening and making music.	0.28	0.47
32_U3	… is creative when making music.	0.31	0.44
25_U3	… plays music with feeling.	0.37	0.42

### Professional Background and Musical Expertise Associated With Differences in the Evaluation of Musicality

Through regression, we computed the component scores for all participants. Subsequently, we explored the influence of profession using a multivariate analysis of variance with professional background (professional educators, parents, and caregivers) as the independent variable and the two component scores as dependent variables. The test revealed no significant relationship between professional educators and parents and carers [*V* = 0.31, *F*(2, 166) = 2.63, *p* = 0.08, partial η*^2^* = 0.031], suggesting that a professional background in child education did not influence the conceptions of musicality in infants/toddlers. However, the correlations between the Gold-MSI musical training subscale and the components Basic Musical Abilities (*r* = 0.26, *p* = 0.001) and Enthusiasm and Motivation (*r* = 0.27, *p* < 0.001) of the MCQ_U3 short scale, were significant and of low to moderate strength, indicating that musical expertise is associated with slightly higher ratings on both components.

### Differences Between Conceptions of Musicality in Infants/Toddlers (<3 Years) and Older Children (3–6 Years)

Inspection of item overlap on the short scales (MCQ vs. MCQ_U3) showed that the component Musical Communication (MCQ) did not have any equivalent in the MCQ_U3. Furthermore, the components Analytical Understanding and Musical Abilities (MCQ) were combined into one component of the MCQ_U3 (Basic Musical Abilities). The component Enthusiasm and Motivation was highly similar in both short scales. The high content similarity was also reflected in a high Rand Index (a measure of similarity between two data clusterings; RI = 0.83, RI adjusted for chance = 0.65). This means that items that were grouped into one component in the version for older children fall mostly into one component in the version for younger children as well.

## Discussion

This study explored the origins of musical behavior in early childhood. To this end, we investigated how German adults conceptualize musicality in children under the age of 3 years. More specifically, we were interested in which facets of the construct musicality are considered important by people with different professional and musical experience and whether these facets coincide with those that can be assessed using common musicality tests. Through PCA analysis, we identified four components of musicality in early childhood: Musical Communication, Enthusiasm and Motivation, Adaptive Expressiveness, and Musical Abilities. Interestingly, only this last component reflects traditional conceptions of musicality that strongly relate to aural abilities (we named this component Musical Abilities), whereas the three remaining components dealt with elements not traditionally captured by musical ability tests. Our results are largely consistent with previous findings on musicality in 3- to 6-year-old children (Buren et al., 2021). Thus, three components in the current study were broadly similar to those in the previous study of musicality in 3- to 6-year-old children (Musical Communication, Enthusiasm and Motivation, Musical Abilities). However, we were also able to show that the facets are not completely alike. Thus, the component Analytical Understanding of Music is not found in the under-3-year-olds. Instead, we found the facet Adaptive Expressiveness, which was not part of musicality in older children.

The Adaptive Expressiveness component was found to represent emotional facets of musicality. These include the ability to perceive and respond to the mood of musical stimuli. The component represents the ways in which children adapt their expressive reactions to different aspects of the music. In addition, items of this component indicate that children who are considered musical are open-minded to all types of music and recognize and appreciate different kinds of music. Analytical Understanding of Music, which was a component of musicality conceptions in older children (Buren et al., 2021), probably does not yet play a role in younger children because of developmental differences. Younger children seem to have a more spontaneous and intuitive access to music, whereas in older children the access to music may have become more analytical and includes a cognitive understanding as well.

On average, enthusiasm for music and the motivation to become musically active was rated by all participants as the most important sign of musicality in children under 3 years of age. This is in line with previous conceptions of child musicality in children older than 3 years (Buren et al., 2021). This suggests that enthusiasm for music is seen as a foundation for musical skill development. Although almost all infants generally show interest in musical stimuli and activities, interindividual differences in musical enthusiasm and motivation could be interpreted as indicators of musicality. These interindividual differences in musical interest might also be linked to the Big Five personality trait openness-to-experience which has been associated with aesthetic interest (McManus and Furnham, 2006) and musical engagement in both children and adults (Corrigall et al., 2013; Müllensiefen et al., 2014). The assumption that motivation predicts musical achievement is also evident in the observation that motivational factors are often used as criteria for selecting musical talent among students (Haroutounian, 2000). Motivation has also been conceptualized as a facet of musicianship in adulthood, recognizing the importance of deliberate effort in the development of musical expertise (Hallam, 2010).

Nevertheless, the measurement of motivational aspects has not yet been given much attention in the scientific assessment of musicality. Perhaps this is the result of methodological difficulties in the measurement of motivational aspects or is a reflection of the concern that motivation may be short-lived (Karma, 2007). However, because enthusiasm and motivation seem to be crucial components of musicality, we need valid measurements, questionnaires, or systematic observations to gain further insight into their importance for musical ability development.

The component Musical Abilities reflects traditional conceptions of musical ability relating to audiation, as assessed by traditional musicality tests. Examples include having a sense of timing and rhythm, a feeling for the beat, and a good musical ear. Additionally, some items describe the ability to move in time with music and find matching gestures and movements. This shows that musicality is not limited to perceptual musical skills, but is rather perceived as diverse and multiform, involving motor skills and the integration of skills across senses. Even in very young children our results demonstrate the complexity of musical behaviors.

The component Musical Communication focuses on using music for communicative purposes. To communicate musically, infants need a considerable “toolbox” for the perception and stimulation of communicative signals (Malloch, 2000). Hence, on the one hand, the component included items on the ability to capture and internalize musical stimuli; on the other hand it included the ability to invent melodies or rhythms, produce musical sounds, rhythmic movements, or sing.

Reducing the items for the short scale of the MCQ_U3 resulted in a 2-component model in which the Enthusiasm and Motivation component remained stable in content and the remaining three components were combined into one component (which we named Basic Musical Abilities). The component Basic Musical Abilities should thus be understood as something significantly more complex than what conventional measures of musical ability capture, which underpins the need for additional measures that cover a broader range of abilities and behaviors.

Using the short scale, we found that professional training in education or professional experience as an educator had no significant effect on the ratings of either component which stands in contrast to previous findings (although participants with more musical training gave slightly higher ratings on both components). In general, our results suggest that there is a high level of agreement about what constitutes musicality in young children.

Adult caregivers shape children’s musical environment and their conception of childhood musicality forms the basis of their musical nurturing and education. Therefore, it is important to understand how adults view children’s musicality, because musical interactions between caregiver and infant contribute to the infant’s musical development in a meaningful way. Our study has shown that parents and carers are aware of motivational aspects being a key component in musical development. It remains unclear if they are aware of ways to foster their children’s motivation. Many parents might be unaware of their own musicality and might rely on recorded music or institutional learning to develop their child’s musical skills (Trehub, 2015).

### Limitations and Future Directions

Finally, potential limitations need to be considered. Our questionnaire was based on statements obtained from a qualitative study of conceptions of musicality in adults (Hallam and Prince, 2003). We conscientiously adjusted the statements to reflect conditions in childhood. However, because the original study focused on musicality in adulthood, the original responses may not have included all aspects of musicality in under-3-year-olds. Nevertheless, the high level of agreement with the selected statements showed that the used items, in general, reflected the conceptions of our sample.

The focus on social conceptions of musicality entails the limitation that the results may be restricted to a German population or Western music cultures. In particular, these conceptions may relate to children’s experiences and opportunities. It has been shown that the musical abilities of infants/toddlers and preschoolers differ significantly and that parents and caregivers perceive this accordingly and adjust their musical endeavors (Politimou et al., 2018). Thus, the environment strongly influences the musical behaviors exhibited and, consequently, the conceptions of musicality. In addition, the selected age range (0–3 years) is associated with large developmental changes. Some items were therefore not necessarily suitable for children across the full age range (some behaviors may not yet be exhibited by the youngest children). Here, a more precise differentiation could provide a clearer picture. Nevertheless, we think that our results are a first step toward capturing social constructs of musicality in very young children. Based on our results, however, finer classifications should be chosen for further studies and test development in order to better reflect developmental aspects.

Another future goal is to use the identified conceptions as a basis for examining childhood musicality and to explore whether this framework can be used to develop and validate test procedures as well as rating scales that reflect the social construct of musicality and, in so doing, trace the nature of musicality.

Hopefully, such test procedures will help to sketch out a more complete and comprehensive picture of musical development. Moreover, they could serve to identify individual differences in musical development between children.

At the moment we tend to draw our conclusions from sample averages rather than individual data (Sloboda, 2008). A key problem of these studies is, however, that their results tell us little about how individual developmental trajectories are shaped, because this information can get lost in aggregated values (Sloboda, 2008).

### Practical Implications

The results of this study illustrate the complexity and multi-layered nature of musicality — even among the very young. Furthermore, we were able to show that conceptions of musicality are differentiated and adapted to musical and general developmental levels. In line with previous studies, the results also point to the great importance of enthusiasm and motivation for musical development processes. Given that everyone has some degree of musical potential (Gordon, 1987; Gembris, 1997; Honing, 2018), what can be done to help children to nurture their individual gifts? There is evidence that a musically enriched environment in which music is appreciated and enjoyed is critical for future development of musical ability (Shuter-Dyson, 1999) and may help children to develop a positive musical self-concept and motivation to develop their skills (Sloboda et al., 1994). The promotion of musical development must therefore not be limited to music lessons, but should explicitly include supposedly “ordinary” activities such as singing with the child (Papoušek, 1982; Trevarthen and Malloch, 2002). The importance of these experiences as learning opportunities may be greatly underestimated (Papoušek, 1982). Parents and caregivers (or educators) can thus promote musicality by interacting musically with children, stimulating their musical interests, and providing them with joyful musical experiences. Hence, early years musical development can be supported by acknowledging the spontaneous musical outputs of young children (e.g., rhythmic movements, cooperative singing) and responding to them. Such interactive forms of musical communication may be very effective in supporting children’s enthusiasm and motivation for music (Flohr and Trevarthen, 2008).

## Conclusion

This study is a first step toward enhancing a general understanding of conceptions of musicality in children under 3 years. We were able to show that already in early childhood, a multifaceted picture of musicality prevails. In particular, adaptive expressiveness, the ability to react flexibly to different musical stimuli, was shown for the first time to be a component of musicality. Furthermore, the results have highlighted the role of enthusiasm and motivation even in the youngest age group and have corroborated earlier findings that conceptions of musicality are broad and multifaceted. This reinforces the notion that traditional musical ability tests can only capture a limited portion of musicality and should therefore be supplemented by new assessment procedures, questionnaires, or observational measurement instruments. In the long run, a more comprehensive view and assessment of musicality may lead to an in-depth understanding of individual musical developmental trajectories and a better means of support for musical development.

## Data Availability Statement

The raw data supporting the conclusions of this article will be made available by the authors, without undue reservation.

## Ethics Statement

The studies involving human participants were reviewed and approved by the Ethics Council of the Max Planck Society. The patients/participants provided their written informed consent to participate in this study.

## Author Contributions

TR and VB contributed to the data collection. VB performed the literature search and drafted the manuscript. VB and DM performed the statistical analysis. FD and DM provided the critical revisions. All authors contributed equally in the project’s conception and design of the questionnaire, contributed to the article, and approved the submitted version.

## Conflict of Interest

The authors declare that the research was conducted in the absence of any commercial or financial relationships that could be construed as a potential conflict of interest.

## Publisher’s Note

All claims expressed in this article are solely those of the authors and do not necessarily represent those of their affiliated organizations, or those of the publisher, the editors and the reviewers. Any product that may be evaluated in this article, or claim that may be made by its manufacturer, is not guaranteed or endorsed by the publisher.
